# HIV Infection-Related Care Outcomes among U.S.-Born and Non-U.S.-Born Blacks with Diagnosed HIV in 40 U.S. Areas: The National HIV Surveillance System, 2016

**DOI:** 10.3390/ijerph15112404

**Published:** 2018-10-30

**Authors:** Hanna B. Demeke, Anna S. Johnson, Hong Zhu, Zanetta Gant, Wayne A. Duffus, Hazel D. Dean

**Affiliations:** 1 Oak Ridge Institute for Science and Education, Office of Health Equity in the Office of the Director at the National Center for HIV/AIDS, Viral Hepatitis, STD, and TB Prevention (NCHHSTP), Centers for Disease Control and Prevention (CDC), Atlanta, GA 30329, USA; 2 Division of HIV/AIDS Prevention (DHAP) at NCHHSTP, CDC, Atlanta, GA 30329, USA; ats5@cdc.gov (A.S.J.); uwf5@cdc.gov (Z.G.); 3 ICF International, DHAP, NCHHSTP, CDC, Atlanta, GA 30329, USA; hongzhu1116@gmail.com; 4 Division of Global HIV and TB, National Center for Global Health, CDC, Atlanta, GA 30329, USA; wed4@cdc.gov; 5 Office of the Director, at NCHHSTP, CDC, Atlanta, GA 30329, USA; hdd0@cdc.gov

**Keywords:** human immunodeficiency virus, acquired immunodeficiency syndrome, African Americans, emigrants and immigrants, health disparity, sustained virologic response, linkage to HIV medical care, retention in medical care, viral suppression, late diagnosis

## Abstract

HIV care outcomes must be improved to reduce new human immunodeficiency virus (HIV) infections and health disparities. HIV infection-related care outcome measures were examined for U.S.-born and non-U.S.-born black persons aged ≥13 years by using National HIV Surveillance System data from 40 U.S. areas. These measures include late-stage HIV diagnosis, timing of linkage to medical care after HIV diagnosis, retention in care, and viral suppression. Ninety-five percent of non-U.S.-born blacks had been born in Africa or the Caribbean. Compared with U.S.-born blacks, higher percentages of non-U.S.-born blacks with HIV infection diagnosed during 2016 received a late-stage diagnoses (28.3% versus 19.1%) and were linked to care in ≤1 month after HIV infection diagnosis (76.8% versus 71.3%). Among persons with HIV diagnosed in 2014 and who were alive at year-end 2015, a higher percentage of non-U.S.-born blacks were retained in care (67.8% versus 61.1%) and achieved viral suppression (68.7% versus 57.8%). Care outcomes varied between African- and Caribbean-born blacks. Non-U.S.-born blacks achieved higher care outcomes than U.S.-born blacks, despite delayed entry to care. Possible explanations include a late-stage presentation that requires immediate linkage and optimal treatment and care provided through government-funded programs.

## 1. Introduction

Late-stage human immunodeficiency virus (HIV) diagnosis, linkage to HIV medical care, retention in HIV medical care, and viral suppression are the core indicators used to monitor progress toward attainment of HIV-related national goals in the United States [1]. Regardless of race, 21.3% of persons with HIV diagnosed during 2016 received a late-stage diagnosis (infection was classified as stage 3 (acquired immunodeficiency syndrome (AIDS)) at the time of diagnosis) [2]. Among persons whose infection was diagnosed in 2016, 75.9% were linked to HIV medical care ≤1 month after diagnosis [2]. Among persons with HIV infection diagnosed by year-end 2014 and who were alive at year-end 2015, 73.4% received any HIV medical care, and 57.2% were retained in HIV medical care during 2015 [2]. Viral load was suppressed among 85.2% of persons with a reported viral load test during 2015 [2].

Blacks or African Americans (hereafter referred to as blacks) are the most affected racial group in the United States. Although blacks comprise only 12% of the U.S. population, they accounted for 44% of HIV diagnoses in 2016 [3]. Improving HIV infection-related care outcomes is crucial for reducing both the number of new HIV infections and health disparities that persist among the U.S. black population [1]. Blacks are more likely to enter HIV infection-related medical care late in the disease course, often with a concurrent AIDS diagnosis [4]. They also are less likely to be linked to medical care [5] and less likely to achieve viral suppression than whites or Hispanics/Latinos [5]. Early detection of HIV infection is essential for timely linkage to medical care and for treatment that will result in immunologic recovery and improved survival.

U.S. residents born outside the United States face multiple challenges related to language, health insurance coverage, and legal status, all of which can affect access to healthcare [6,7,8]. Differences exist in health-related behaviors, physical and sociocultural environments, and usage of healthcare systems between U.S.-born and non-U.S.-born persons [7]. These differences can exacerbate negative health outcomes at the individual, interpersonal, community, and societal levels [8]. Non-U.S.-born persons with HIV infection can experience discrimination based on syndemic factors, including socioeconomic disadvantages and immigration-related legal problems in addition to their HIV infection [9,10].

During 2008–2014, 11.1% of black U.S. residents with diagnosed HIV were born outside the United States [11]. Research regarding HIV infection-related care outcomes has mainly been conducted among the overall black population and has not described outcomes for non-U.S.-born blacks at the national level. Combining all blacks into one group masks differences that can exist between U.S.-born and non-U.S.-born blacks that are crucial for understanding health disparities and inequities among the U.S. black population [12].

Non-U.S.-born blacks have higher annual HIV infection diagnosis rates and a higher percentage of persons who receive a late-stage HIV diagnosis, compared with U.S.-born blacks, although the survival rate among non-U.S.-born blacks is greater [11,13]. Studies have been needed that assess why non-U.S.-born blacks have higher survival rates after receiving an HIV diagnosis, even though higher percentages of non-U.S.-born blacks received initial diagnoses at a late disease stage. Comparing HIV care outcomes among these groups at the national level might explain in part the existence of this paradox as the care provided or received after diagnosis of HIV; for example, linkage to care and retention in care can determine the person’s survival. Our paper examines the distribution of HIV care outcomes among U.S.-born and non-U.S.-born blacks, specifically, late diagnosis, linkage to HIV infection-related medical care after HIV diagnosis, retention in care, and viral suppression.

## 2. Methods

### 2.1. Data Collection and Classification

The Centers for Disease Control and Prevention (CDC, an agency of the U.S. Department of Health and Human Services) collects data through the National HIV Surveillance System (NHSS) in collaboration with state and local health departments. We analyzed data regarding blacks aged ≥13 years who resided in 40 U.S. jurisdictions that reported complete CD4^+^ T-lymphocytes (CD4) and viral load (VL) test results to CDC through December 2017. Jurisdictions that met the criteria for inclusion in our study were those with laws or regulations that required reporting of all CD4 and VL test results to the state or local health department and that had reported to CDC ≥95% of all laboratory test results received during January 2015–September 2017 to allow time for delays in reporting of data to CDC. The 40 U.S. areas were Alabama, Alaska, California, Colorado, Connecticut, Delaware, the District of Columbia, Florida, Georgia, Hawaii, Illinois, Indiana, Iowa, Louisiana, Maine, Maryland, Massachusetts, Michigan, Minnesota, Mississippi, Missouri, Montana, Nebraska, New Hampshire, New Mexico, New York, North Carolina, North Dakota, Oregon, Rhode Island, South Carolina, South Dakota, Tennessee, Texas, Utah, Virginia, Washington, West Virginia, Wisconsin, and Wyoming.

We categorized persons born in the United States or its six dependencies (American Samoa, Guam, Northern Mariana Islands, Puerto Rico, Republic of Palau, and the U.S. Virgin Islands) as U.S.-born and those born outside the United States and its six dependencies as non-U.S.-born [14]. NHSS uses the variable “country of birth” to collect information on birthplace. For geographic analysis, we used the *United Nations Demographic Yearbook*, *2014*, to group countries of birth by world region [15]. The U.S. Census Bureau’s classification of urban and rural areas was used for our study [16], and we classified cases according to the person’s area of U.S. residence at the time of HIV infection diagnosis. Geographic areas with ≥500,000 population were classified as metropolitan; areas with 50,000–499,999 population as urban; and areas with <50,000 population as rural [16].

### 2.2. HIV Infection-Related Care Outcome Measures Defintions

Late-stage diagnosis: A CD4 count of <200/µL, the CD4 percentage of total lymphocytes of <14, or documentation of an AIDS-defining condition at the time of initial HIV diagnosis.

Linkage to HIV infection-related medical care: Documentation of ≥1 CD4 count or percentage, or VL tests performed <1 month or 1–<3 months after HIV infection diagnosis. Those with no documented CD4 or VL tests through December 2017 were classified as not linked to care.

Retention in HIV infection-related medical care: Documentation of ≥2 CD4 or VL tests performed ≥3 months apart during 2015.

Viral suppression: VL of <200 copies/mL at the last test during 2015.

### 2.3. Statistical Analysis

We examined distributions of HIV diagnoses by country of birth, selected demographics, and HIV-transmission risk factors for all HIV diagnoses regardless of person’s stage of diseases, and for persons who received a late-stage diagnosis. We also assessed the time between HIV diagnosis and the first documentation of CD4 or VL laboratory result and examined distributions by country of birth, selected demographics, and HIV-transmission risk factors for persons linked to HIV infection-related medical care <1 month, 1–<3 months, >3 months, and no documented linkage through December 2017. Late-stage diagnosis and linkage to HIV infection-related medical care were assessed among blacks aged ≥13 years who had received an HIV infection diagnosis during 2016 and who resided in one of the 40 jurisdictions at the time of diagnosis.

Next, we examined retention in HIV medical care and viral suppression among blacks aged ≥13 years who had received an HIV infection diagnosis during 2014; were alive as of December 31, 2015; and who resided in one of the 40 jurisdictions at the time of diagnosis. Our analysis did not include those who had received an HIV infection diagnosis before 2014. To allow 24 months for delays in reporting of deaths, data from the most recent year (2016) were excluded.

Statistical tests to determine the significance of observed differences were not completed because standard errors of those differences were unavailable. Computing standard errors would have required unreasonable assumptions about the correct theoretical sampling distribution.

## 3. Results

### 3.1. HIV Infection Diagnoses

A total of 15,021 blacks aged ≥13 years received a diagnosis of HIV infection during 2016 in 40 U.S. jurisdictions, of whom 11,185 (74.5%) had place-of-birth information reported to CDC. Of those with complete place-of-birth information, 1744 (15.6%) had been born outside the United States or its territories. Among non-U.S.-born blacks, 974 (55.8%) had been born in Africa, and 673 (38.6%) in the Caribbean.

Approximately half (53.5%) of non-U.S.-born blacks who had received an HIV infection diagnosis during 2016 were female, compared with 21.8% of U.S.-born blacks (Table 1). The age distribution also differed, with blacks aged ≥35 years accounting for a higher percentage (66.3%) of non-U.S.-born blacks with an HIV diagnosis during 2016, compared with U.S.-born (35.0%) blacks. Among non-U.S.-born blacks, HIV diagnoses attributed to heterosexual contact accounted for the highest percentage of diagnoses among both males (49.0%) and females (94.9%). For U.S.-born blacks, HIV diagnoses attributed to male-to-male sexual contact accounted for the highest percentage (79.9%) of diagnoses among males, whereas HIV infection diagnoses attributed to heterosexual contact accounted for the highest percentage (90.5%) among females. The percentage of HIV infection diagnoses attributed to male-to-male sexual contact among non-U.S.-born black males was lower than that among U.S.-born black males (46.5% versus 79.9%, respectively). The percentage of HIV infection diagnoses attributed to injection-drug use among non-U.S.-born black females was lower than among U.S.-born black females (3.4% versus 9.1%, respectively) (Table 1). A higher percentage (89.4%) of non-U.S.-born blacks, compared with 79.1% of U.S.-born blacks lived in metropolitan areas (Table 1).

### 3.2. Late-Stage HIV Diagnosis (Stage 3 (AIDS) Classification at the Time of Diagnosis of HIV Infection)

Of 11,185 blacks aged ≥13 years with HIV infection diagnosed during 2016 throughout the 40 jurisdictions and had place-of-birth information reported to CDC, 2300 (20.6%) received a late diagnosis (28.3% of non-U.S.-born and 19.1% of U.S.-born blacks) (Table 1). A higher percentage of non-U.S.-born black males received a late-stage diagnosis, compared with non-U.S.-born black females (33.2% male versus 24% female) at the time of diagnosis; in contrast, a higher percentage of females received a late-stage diagnosis among U.S.-born blacks (18.5% male versus 21.5% female). Receipt of late-stage HIV infection diagnoses increased as age increased for U.S.-born blacks. Among non-U.S.-born blacks, persons aged 35–44 years accounted for the highest percentage of blacks who received a late-stage diagnosis (34.4%). The percentages of blacks who received a late-stage diagnosis during 2016 were similar for persons who had been born in Africa and the Caribbean (28.1% and 27.8%, respectively) (Table 1).

### 3.3. Linkage to HIV Infection-Related Medical Care

**Figure 1:**
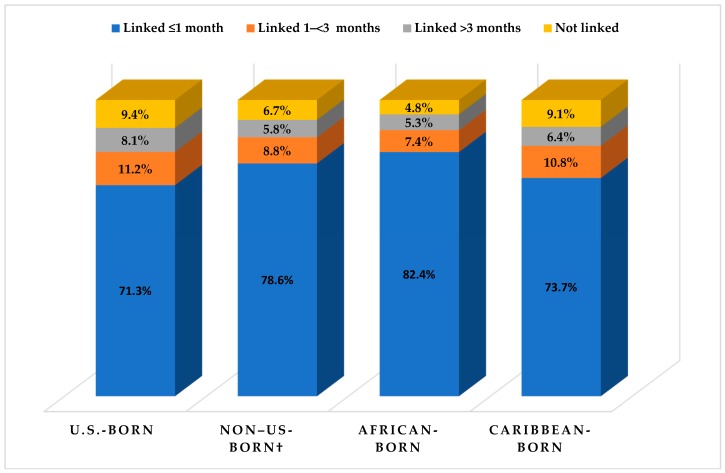
Linkage to HIV infection-related medical care among blacks aged ≥13 years with HIV infection diagnosed during 2016, by place of birth, National HIV Surveillance System, 40 U.S. areas. † Includes African- and Caribbean-born blacks.

Among non-U.S.-born blacks aged ≥13 years with HIV infection diagnosed during 2016 in the 40 jurisdictions and who had place-of-birth information reported to CDC, 78.6% had been linked to care in ≤1 month after diagnosis, compared with 71.3% of U.S.-born blacks; 6.7% had not been linked by December 2017 after receiving a diagnosis, compared with 9.4% of U.S.-born blacks (Table 2). Among non-U.S.-born blacks with a late-stage diagnosis, 90.5% had been linked to care in ≤1 month after diagnosis, compared with 89.7% of U.S.-born blacks with a late-stage diagnosis; 0.6% had not been linked after receiving a late-stage diagnosis, compared with 0.1% of U.S.-born blacks (Table 2). A lower percentage of non-U.S.-born blacks had not been linked to HIV infection-related medical care in all selected demographic characteristics, except for black males with HIV infection attributed to male-to-male sexual contact and injection-drug use (12.5% of non-U.S.-born versus 11.1% of U.S.-born blacks) and rural black residents (6.7% of non-U.S.-born versus 5.5% of U.S.-born blacks). When we assessed care outcomes among non-U.S.-born blacks by world region of birth, the percentage of blacks linked to care in ≤1 month among Caribbean-born (73.3%) and U.S.-born blacks (71.3%) were lower than 82.4% of African-born blacks linked to care in ≤1 month. U.S.-born (9.4%) and Caribbean-born (9.1%) blacks had higher percentages not linked to care, compared with African-born blacks (4.8%) (Table 2 and Figure 1).

### 3.4. Retention and Viral Suppression

A total of 14,799 blacks aged ≥13 years received a diagnosis of HIV infection during 2014 and were alive at year-end 2015 in the 40 jurisdictions, of whom 11,742 (79.3%) had place-of-birth information reported to CDC. Of those with complete place-of-birth information, 1542 (13.1%) had been born outside the United States. Among non-U.S.-born persons, 899 (58.3%) had been born in Africa, and 565 (36.6%) had been born in the Caribbean. Table 3 presents the demographic characteristics and the percentages of blacks who received a late-stage HIV diagnosis. Of 11,742 blacks aged ≥13 years with HIV infection diagnosed during 2014 in the 40 jurisdictions, 7274 (61.9%) had been retained in care, and 6957 (59.2%) had achieved viral suppression during 2015 (data not shown).

**Figure 2:**
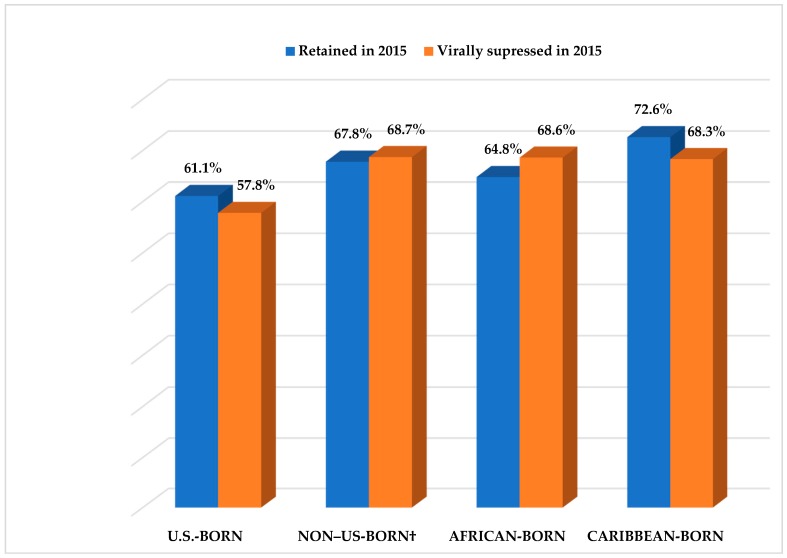
Retention and viral suppression during 2015 among blacks aged ≥13 years with HIV infection diagnosed during 2014 and who were alive as of December 31, 2015, by place of birth, National HIV Surveillance System, 40 U.S. areas. † Includes African- and Caribbean-born blacks.

Among non-U.S.-born blacks, 67.8% had been retained in care, and 68.7% had achieved viral suppression by year-end 2015, compared with 61.1% and 57.8% of U.S.-born blacks, respectively (Table 3 and Figure 2). A higher percentage of non-U.S.-born blacks had been retained in care and had achieved viral suppression during 2015 than U.S.-born blacks, when the data were assessed by selected demographic characteristics, except those aged ≥55 years and persons residing in rural areas at the time of diagnosis.

The percentages of non-U.S.-born blacks who had achieved viral suppression were higher than the percentages retained in care for the majority of the selected demographic characteristics, except those aged 13–24 years and ≥55 years and females with HIV transmission categorized as “other.” In contrast, among U.S.-born blacks, the percentage who had achieved viral suppression was consistently lower than the percentage retained in care across all demographic characteristics.

When non-U.S.-born blacks were further categorized by world region of birth, 72.6% of Caribbean-born blacks had been retained in care during 2015, compared with 64.8% of African-born and 67.9% among those who had been born in world regions other than Africa or the Caribbean. However, 71.8% of blacks born in world regions other than Africa or the Caribbean had achieved viral suppression, compared with 68.6% and 68.3% of African- and Caribbean-born blacks, respectively (Table 3). The percentage of Caribbean-born blacks who had achieved viral suppression (68.3%) was lower than the percentage retained in care (72.6%). Unlike Caribbean-born blacks, a higher percentage of African-born (68.6%) and non-U.S.-born blacks born in other world regions (71.8%) had achieved viral suppression, compared with African-born blacks (64.8%) and non-U.S.-born blacks born in other world regions (67.9%) who had been retained in care (Table 3 and Figure 2).

## 4. Discussion

Our findings demonstrate that variations exist in late-stage HIV diagnosis, linkage to and retention in HIV infection-related medical care, and viral suppression between non-U.S.-born and U.S.-born blacks. These findings are important for understanding and ensuring HIV infection-related care equity among blacks residing in the United States and provides empirical evidence that blacks with diagnosed HIV are not a homogeneous group. Despite late presentation into HIV infection-related medical care, higher percentages of non-U.S.-born blacks are linked to and retained in care and are virally suppressed, which supports previous findings of higher survival among non-U.S.-born blacks [11,13]. Details regarding the epidemiologic and demographic differences between non-U.S.-born and U.S.-born blacks with diagnosed HIV by using NHSS data are available elsewhere [11,13].

Similar to previous reports, in our study, 95% of non-U.S.-born blacks were from Africa or the Caribbean regions and were more likely to receive a late-stage HIV diagnosis [11,13]. Multiple barriers that delay HIV testing among African- and Caribbean-born blacks have been reported, including socioeconomic disadvantages, immigration-related barriers, and fears of stigma [17,18]. However, higher percentage of non-U.S.-born blacks in this study were linked to HIV infection-related medical care, compared with their U.S.-born counterparts. This finding was not anticipated because non-U.S.-born populations typically have been disproportionally lacking health coverage and receive fewer medical services than U.S.-born persons [8]. An examination of healthcare coverages demonstrated that a substantially higher proportion of non-U.S.-born persons were uninsured and received Ryan White HIV/AIDS Program (RWHAP) assistance, compared with U.S.-born persons receiving HIV infection-related medical care [19]. RWHAP provides services for persons living with HIV who are uninsured [20] and offsets the lack of healthcare coverage that would otherwise have hindered linkage to HIV care and treatment. Another possible reason for better linkage among non-U.S.-born blacks, of whom a higher proportion received a late-stage diagnosis, is symptomatic illness at the time of presentation for care requiring immediate linkage to care and treatment [21].

African-born blacks accounted for the higher percentage of persons linked to care in ≤1 month after diagnosis and the lowest percentage not linked to care, compared with Caribbean-born blacks. African-born blacks were more likely to receive a diagnosis and have no differences in late-stage HIV diagnosis, compared with Caribbean-born blacks, according to previous studies [11]. African-born blacks with diagnosed HIV were more likely to be female (60.7% versus 44.7%) and aged 25–44 years (63.3% versus 47.7%) than Caribbean-born blacks [11]; thus, they might be more likely to have healthcare access primarily for seeking pregnancy– and child-bearing–related care. Previous studies indicated a substantially higher proportion of African-born black women who received pregnancy care, compared with Caribbean-born black women. For example, 39% of women who became pregnant after receiving an HIV diagnosis in Rhode Island (USA) during 2004–2009 had been born in Africa, compared with only 3% who had been born in the Caribbean [22]. Another multisite longitudinal study that compared U.S.-born and non-U.S.-born black mothers with HIV infection reported that 23% of HIV-exposed infants had been born to non-U.S.-born black mothers, of whom 65% were African-born, compared with 25% of mothers who had been born in the Caribbean [23].

Retention in HIV medical care was higher among non-U.S.-born blacks, compared with their U.S.-born counterparts. The availability of RWHRP assistance might not only alleviate the healthcare coverage challenges of non-U.S.-born blacks as discussed earlier, but it might also provide for optimal medical and supportive care that is essential for retention [24,25]. Although both African and Caribbean-born blacks had higher percentages of retention than U.S.-born blacks, Caribbean-born blacks were retained in care at a higher percentage than African-born blacks. Caribbean-born persons are more likely than African-born persons to have lived in the U.S. longer and to speak proficient English [26]; thus, Caribbean-born persons might have greater social support and are more likely to be able to navigate the healthcare system. Persons with limited English proficiency often have trouble obtaining specific healthcare because of the paucity of translated medical materials and the lack of trained medical interpreters or bilingual providers [18]. Possibly, African-born persons are less likely to maintain a regular visit to HIV medical care to avoid disclosure of status because of fear of HIV infection-related stigma. HIV infection-related stigma is strong among African-born black communities and has been associated with reluctance to get tested and with lack of retention in the HIV infection-related medical care [27].

In our study, non-U.S.-born blacks achieved viral suppression at a higher percentage than U.S.-born blacks. However, previous reports on viral suppression are mixed. In one study, non-U.S.-born persons in the Caribbean region achieved almost similar viral suppression, compared with U.S.-born persons (73.4% versus 73.3%) and lower viral suppression, compared with non-U.S.-born persons from Africa (73.4% versus 78.3%) [19]. Another study reported substantial differences between U.S.-, African-, and Caribbean-born persons with HIV infection in 2009 by using a national representative sample in which Caribbean-born persons achieved substantially less viral suppression than African- or U.S.-born persons [28]. Both studies, however, included white and Hispanics in their study, which limited our ability to do a direct comparison with our findings.

Despite the lower rate of retention in HIV infection-related medical care than Caribbean-born blacks, African-born blacks achieved slightly higher viral suppression than Caribbean-born blacks. Our study demonstrates that lower retention among African-born blacks does not translate into correspondingly poorer achievement of viral suppression. One explanation might be the way retention in HIV infection-related medical care was defined. An African-born person might have reduced the frequency of clinical encounters and spaced their visits to the HIV provider because of fear of disclosure [27], although they might not necessarily have skipped treatment or other needed services that support viral suppression achievement. Persons who had only one visit during 2015 were not counted as retained because retention in HIV infection-related medical care is defined as ≥2 CD4 or VL tests performed during 2015. Further investigations are needed because achievement of viral suppression is associated with multiple factors (e.g., the regularity of clinic visits, prescription and adherence to antiretroviral therapy, and presence of other comorbidities, including mental health and substance use).

Our study is subject to certain limitations. NHSS data might not represent all U.S.-born and non-U.S.-born blacks with diagnosed HIV infection in the United States for two reasons. First, this study is limited to 40 public health jurisdictions and persons whose place of birth was reported. Second, not all persons with HIV infection are identified and their information entered into the NHSS database, including those who are unaware of their HIV status; however, NHSS represents 85% of persons with HIV infection in the United States [29]. Thus, we were unable to assess whether differences in retention in care and vial suppression were related to differences in current residence or duration of residency in the United States after HIV diagnosis. Furthermore, documentation of retention and viral load for 1 year only might not be indicative of consistent retention and viral suppression over time. Although NHSS collects selected treatment data, it is limited and not collected consistently across reporting jurisdictions. Thus, we were unable to assess whether differences in viral suppression were associated with differences in uptake of HIV treatment.

## 5. Conclusions

Differential access and usage of HIV infection-related medical care is associated with disparities in health outcomes and is important for guiding improvements in the quality of care and life among U.S.-born and non-U.S.-born blacks with diagnosed HIV infection. Despite higher proportion HIV testing being indicated by lower percentage of late-stage diagnoses, U.S.-born blacks had lower percentage linked and retained in care and lower percentages were virally suppressed, compared with non-U.S.-born blacks. Consequently, merging U.S.-born and non-U.S.-born blacks into one group inhibits our ability to understand and design needed HIV prevention and control programs. Additional studies can be useful for describing the protective characteristics of non-U.S.-born blacks that lead to better linkage, retention, and viral suppression, despite late presentation to HIV infection-related medical care. Programs designed to improve timely testing need to target non-U.S.-born blacks to achieve improved HIV infection outcomes. HIV prevention and treatment programs that target the U.S. black population need to consider this variation between non-U.S.-born and U.S.-born blacks.

## Figures and Tables

**Table 1:** Late-stage human immunodeficiency virus (HIV) diagnosis (Stage 3, acquired immunodeficiency syndrome (AIDS) at the time of diagnosis) among U.S.-born and non-U.S.-born blacks aged ≥13 years by selected demographic characteristics, National HIV Surveillance System, 40 U.S. areas, 2016.

Characteristic	U.S.-Born				Non-U.S.-Born			
	Total No. of Cases	Col%	Late-Stage Diagnosis ^a^		Total No. of Cases	Col%	Late-Stage Diagnosis ^a^	
			No.	%			No.	%
Total	9441	100	1807	19.1	1744	100	493	28.3
Sex								
Male	7386	78.2	1366	18.5	807	46.3	268	33.2
Female	2055	21.8	441	21.5	937	53.7	225	24.0
Age (years)								
13‒24	2839	30.1	277	9.8	143	8.2	25	17.5
25–34	3291	34.9	543	16.5	445	25.5	96	21.6
35–44	1364	14.4	359	26.3	509	29.2	175	34.4
44‒54	1118	11.8	343	30.7	361	20.7	110	30.5
≥55	829	8.8	285	34.4	286	16.4	87	30.4
Transmission category (male) ^b^								
Male-to-male sexual contact	5899	79.9	994	16.9	376	46.5	99	26.3
Injection-drug use (IDU)	264	3.6	58	22.0	26	3.2	13	50.0
Male-to-male sexual contact and IDU	208	2.8	42	20.2	8	1.0	4	50.0
Heterosexual contact ^c^	1005	13.6	267	26.6	396	49.0	151	38.1
Other ^d^	10	0.1	4	40.0	2	0.2	1	50.0
Transmission category (female) ^b^								
IDU	188	9.1	51	27.1	32	3.4	8	25.0
Heterosexual contact ^c^	1860	90.5	388	20.9	889	94.9	214	24.1
Other ^d^	7	0.3	2	28.6	16	1.7	3	18.8
Type of residence at the time of diagnosis								
Metropolitan (population ≥500,000 persons)	7472	79.1	1348	18.0	1559	89.4	444	28.5
Urban (population 50,000–499,999 persons)	1521	16.1	343	22.6	150	8.6	36	24.0
Rural (population ≤49,999 persons)	384	4.1	104	27.1	30	1.7	11	36.7
Unknown	64	0.7	12	18.8	5	0.3	2	40.0
World region of birth								
Africa	—	—	—	—	974	55.8	274	28.1
Caribbean	—	—	—	—	673	38.6	187	27.8
Other	—	—	—	—	97	5.6	32	33.0

^a^ A CD4 count of <200/µL, the CD4 percentage of total lymphocytes of <14, or documentation of an AIDS-defining condition at the time of initial HIV diagnosis. ^b^ Data have been statistically adjusted to account for missing transmission category. ^c^ Heterosexual contact with a person known to have or to be at high risk for HIV infection. ^d^ Includes hemophilia, blood transfusion, perinatal exposure, and risk factor not reported or not identified.

**Table 2:** Linkage to human immunodeficiency virus (HIV) infection-related medical care among U.S.-born and non-U.S.-born blacks aged ≥13 years, by selected demographic characteristics, National HIV Surveillance System, 40 U.S. areas, 2016.

Characteristic	Percentage of Persons with ≥1 CD4^+^ Count or Viral Load Test After HIV Diagnosis									
	U.S.-Born					Non-U.S.-Born				
	No.	≤1 month	1–≤3 months	>3 months	Not Linked	No.	≤1 month	1–≤3 months	>3 months	Not Linked
Total	9441	71.3	11.2	8.1	9.4	1744	78.6	8.8	5.8	6.7
Sex										
Male	7386	70.4	11.5	8.4	9.6	807	77.8	8.7	5.9	7.6
Female	2055	74.5	9.9	7.0	8.6	937	79.3	9.0	5.8	6.0
Age (years)										
13‒24	2839	69.5	12.6	9.5	8.4	143	77.6	11.2	6.3	4.9
25–34	3291	70.4	11.6	8.9	9.1	445	80.7	7.2	4.7	7.4
35–44	1364	71.6	10.1	7.4	10.9	509	80.2	9.2	5.5	5.1
44‒54	1118	75.1	8.7	4.9	11.3	361	75.1	10	7.2	7.8
≥55	829	75.3	9.7	5.7	9.4	286	77.6	8.0	6.3	8.0
Transmission category (male) ^a^										
Male-to-male sexual contact	5899	70.8	11.6	8.5	9.0	376	76.9	9.6	5.1	8.2
Injection-drug use (IDU)	264	72.0	7.2	6.1	14.8	26	84.6	3.8	3.8	7.7
Male-to-male sexual contact and IDU	208	70.7	14.4	3.8	11.1	8	87.5	12.5	0	12.5
Heterosexual contact ^b^	1005	67.4	11.6	9.4	11.5	396	77.8	8.1	7.1	6.8
Other ^c^	10	70	0	10	10	2	100	0	0	0
Transmission category (female) ^a^										
IDU	188	75.0	8.5	8.0	9.0	32	81.3	9.4	3.1	6.3
Heterosexual contact ^b^	1860	74.4	10.1	6.9	8.6	889	78.9	9.1	6.0	6.1
Other ^c^	7	85.7	0	0	14.3	16	100	0	0	0
Type of residence at the time of diagnosis										
Metropolitan (population ≥500,000 persons)	7472	66.6	10.6	8.2	9.5	1559	78.2	9.0	6.0	6.8
Urban (population 50,000–499,999)	1521	66.7	13.2	8.0	9.9	150	82.7	7.3	4.0	6.0
Rural (population ≤49,999 persons)	384	76.4	14.3	6.5	5.5	30	80	6.7	6.7	6.7
Unknown	64	60.9	10.9	15.6	12.5	5	80	20	0	0
World region of birth										
Africa	—	—	—	—	—	974	82.4	7.4	5.3	4.8
Caribbean	—	—	—	—	—	673	73.7	10.8	6.4	9.1
Other	—	—	—	—	—	97	74.2	9.3	7.2	9.3
Late-stage diagnosis ^d^	1807	89.7	10	0.3	0.1	493	90.5	7.7	1.2	0.6

^a^ Data have been statistically adjusted to account for missing transmission category. ^b^ Heterosexual contact with a person known to have or to be at high risk for HIV infection. ^c^ Includes hemophilia, blood transfusion, perinatal exposure, and risk factor not reported or not identified. ^d^ A CD4 count of <200/µL, the CD4 percentage of total lymphocytes of <14, or documentation of an AIDS-defining condition at the time of initial HIV diagnosis.

**Table 3:** Retention in human immunodeficiency virus (HIV) infection-related medical care and viral suppression during 2015, among U.S.-born and non-U.S.-born blacks aged ≥13 years, with HIV infection diagnosed during 2014 and who were alive at end-of-year 2015, by selected demographic characteristics, National HIV Surveillance System, 40 U.S. areas.

Characteristic	U.S.-Born				Non-U.S.-Born			
	No.	Col%	Retained ^a^ (%)	Virally Suppressed (%)	No.	Col%	Retained ^a^ (%)	Virally Suppressed ^a^ (%)
Total	10,200	100	61.1	57.8	1542	100	67.8	68.7
Sex								
Male	7891	77.4	59.7	57.2	645	41.8	68.1	68.2
Female	2309	22.6	65.7	60.1	897	58.2	67.7	69.0
Age (years)								
13‒24	3097	30.4	59.0	53.6	134	8.7	67.2	59.7
25–34	3413	33.5	59.6	57.5	401	26.0	69.1	70.6
35–44	1572	15.4	62.8	60.9	457	29.6	67.2	71.1
44‒54	1287	12.6	64.4	60.9	298	19.3	70.1	70.8
≥55	831	8.1	66.2	64.3	252	16.3	64.7	63.5
Transmission category (male) ^b^								
Male-to-male sexual contact	6355	80.5	60.5	58.0	298	46.3	67.8	70.5
Injection-drug use (IDU)	293	3.7	55.3	52.2	28	4.3	60.7	67.9
Male-to-male sexual contact and IDU	239	3.0	56.9	47.3	7	1.1	71.4	57.1
Heterosexual contact ^c^	994	12.6	56.7	56.0	307	47.7	68.7	66.4
Other ^d^	11	0.1	45.5	36.4	4	0.6	50.0	75.0
Transmission category (female) ^b^								
IDU	233	10.1	63.1	55.8	34	3.8	61.8	64.7
Heterosexual contact ^c^	2070	89.6	65.9	60.6	852	95.0	68.0	69.4
Other ^d^	6	0.3	50.0	50.0	11	1.2	63.6	54.5
Type of residence at the time of diagnosis								
Metropolitan (Population ≥500,000 persons)	7781	76.3	60.1	57.2	1366	88.6	67.2	67.8
Urban (Population 50,000–499,999)	1565	15.3	62.8	58.1	112	7.3	74.1	77.7
Rural (Population ≤49,999)	385	3.8	67.3	61.6	15	1.0	66.7	66.7
Unknown	469	4.6	65.2	63.5	49	3.2	71.4	73.5
World region of birth								
Africa	—	—	—	—	899	58.3	64.8	68.6
Caribbean	—	—	—	—	565	36.6	72.6	68.3
Other	—	—	—	—	78	5.1	67.9	71.8
Late-stage diagnosis ^e^	2011	19.7	78.4	71.2	416	27.0	77.4	73.8

^a^ During 2015. ^b^ Data have been statistically adjusted to account for missing transmission category. ^c^ Heterosexual contact with a person known to have or to be at high risk for HIV infection. ^d^ Includes hemophilia, blood transfusion, perinatal exposure, and risk factor not reported or not identified. ^e^ A CD4 count of <200/µL, the CD4 percentage of total lymphocytes of <14, or documentation of an AIDS-defining condition at the time of initial HIV diagnosis.
